# Carbon Monoxide Inhibits Tenascin-C Mediated Inflammation via IL-10 Expression in a Septic Mouse Model

**DOI:** 10.1155/2015/613249

**Published:** 2015-10-07

**Authors:** Md. Jamal Uddin, Chun-shi Li, Yeonsoo Joe, Yingqing Chen, Qinggao Zhang, Stefan W. Ryter, Hun Taeg Chung

**Affiliations:** ^1^School of Biological Sciences, University of Ulsan, Ulsan 680-749, Republic of Korea; ^2^Department of Pharmacology, Dalian University Medical college, Dalian, Liaoning 133000, China; ^3^Joan and Sanford I. Weill Department of Medicine, New York-Presbyterian Hospital, and Division of Pulmonary and Critical Care Medicine, Weill Cornell Medical Center, New York, NY 10065, USA

## Abstract

Tenascin-C (TN-C), an extracellular matrix (ECM) glycoprotein, is specifically induced upon tissue injury and infection and during septic conditions. Carbon monoxide (CO) gas is known to exert various anti-inflammatory effects in various inflammatory diseases. However, the mechanisms underlying the effect of CO on TN-C-mediated inflammation are unknown. In the present study, we found that treatment with LPS significantly enhanced TN-C expression in macrophages. CO gas, or treatment with the CO-donor compound, CORM-2, dramatically reduced LPS-induced expression of TN-C and proinflammatory cytokines while significantly increased the expression of IL-10. Treatment with TN-C siRNA significantly suppressed the effects of LPS on proinflammatory cytokines production. TN-C siRNA did not affect the CORM-2-dependent increase of IL-10 expression. In cells transfected with IL-10 siRNA, CORM-2 had no effect on the LPS-induced expression of TN-C and its downstream cytokines. These data suggest that IL-10 mediates the inhibitory effect of CO on TN-C and the downstream production of proinflammatory cytokines. Additionally, administration of CORM-2 dramatically reduced LPS-induced TN-C and proinflammatory cytokines production while expression of IL-10 was significantly increased. In conclusion, CO regulated IL-10 expression and thus inhibited TN-C-mediated inflammation *in vitro* and *in vivo*.

## 1. Introduction

Damage-associated molecular patterns (DAMPs) are endogenous molecules that can perpetuate inflammatory responses during cell stress or injury. The ECM glycoprotein TN-C, is specifically induced upon tissue injury [1, 2] and infection [3, 4] and upregulated in septic patients [5]. TLR4-mediated TN-C expression induces cytokine production in both human and murine macrophages and in rheumatoid arthritis synovial fibroblasts [6]. Activated TLR4 induces TN-C expression in synovial fibroblasts and myeloid cells [7]. TN-C induction is crucial for the proinflammatory response* in vivo* [8]. Importantly, glucocorticoids can inhibit the expression of TN-C in bone marrow stromal cells and fibroblasts [9]. In addition, mice and bone marrow-derived macrophages (BMDMs) deficient in TN-C display lower production of proinflammatory cytokines such as TNF-*α* during LPS-induced sepsis. Thus, TN-C has been recognized as a regulator of the early immune response [8].

IL-10 is a vital anti-inflammatory cytokine which is required for dampening inflammatory signals and defending the host from excessive inflammation [10]. Mice lacking IL-10 infected with bacterial pathogens display high mortality, associated with excessive inflammatory responses [10]. Low levels of IL-10 expression were associated with various inflammatory diseases such as ulcerative colitis, Crohn's disease, and asthma in humans [11, 12]. The anti-inflammatory effect of IL-10 is mediated through the JAK1-STAT3 pathway which leads to the inhibition of proinflammatory proteins such as TNF-*α* and IL-6 [10, 13]. Higher expression of IL-10 was found in BMDMs from TN-C-deficient mice while there was lower expression of proinflammatory cytokines [8], indicating an anti-inflammatory role of IL-10 in the TN-C-mediated inflammatory disease model.

Carbon monoxide (CO) is generated as an end product of the oxidative degradation of heme by the enzymatic action of heme oxygenase, which converts heme into biliverdin, free iron, and CO [14]. Anti-inflammatory effects of CO have been evident in murine models of sepsis, postoperative ileus, and organ xenotransplantation [15, 16]. In addition, CO has been found to be an important regulator in the suppression of inflammatory cytokines and mediators including inducible nitric oxide synthase (iNOS), TNF-*α*, and IL-6 [17, 18] as well as induction of the anti-inflammatory cytokine IL-10 [19]. The production of endogenous CO was essential for IL-10-dependent inhibition of TNF-*α* expression [20]. To date, there are no reports regarding the effects of CO-mediated IL-10 production on the regulation of TN-C-mediated inflammation. Therefore, in the current study, we examined the effects of CO-dependent IL-10 generation on TN-C-mediated inflammation in macrophages and in the septic mice model.

## 2. Methods and Materials

### 2.1. Reagents and Antibodies

Tenascin-C antibody was purchased from Cell Signaling Technology (MA, USA). *β*-actin, anti-mouse and anti-goat antibodies conjugated to horseradish peroxidase were obtained from Santa Cruz Biotechnology (Santa Cruz, CA, USA). Lipopolysaccharide (LPS) and protease inhibitor cocktail sets were purchased from Sigma-Aldrich (St. Louis, MO, USA). Zinc protoporphyrin IX (ZnPPIX) was obtained from Frontier Scientific Inc. Recombinant mouse IL-10 proteins were purchased from R&D systems. Dulbecco's Modified Eagle Medium (DMEM), fetal bovine serum (FBS), penicillin-streptomycin, and sodium pyruvate were purchased from Invitrogen (Grand Island, NY, USA). All other chemicals were obtained from Sigma-Aldrich.

### 2.2. Cell Culture

RAW 264.7 cells and peritoneal macrophages were cultured in DMEM (Invitrogen) containing 10% Fetal Bovine Serum (FBS) and 1% penicillin streptomycin at 37°C in 5% CO_2_ until 75–80% confluence. For preparation of peritoneal macrophages, mice were injected intraperitoneally with 3% thioglycolate for 3 days and cells were collected for culture. Cells (5 × 10^5^/mL) were seeded in 6-well plates and incubated overnight for subsequent experiments.

### 2.3. Animal Model

Seven-week-old wild type male C57BL/6 mice were pretreated with CORM-2 (30 mg/kg, i.p.) [21] or RuCl_3_ (30 mg/kg, i.p.). And then, mice were injected with LPS (10 mg/kg, i.p.). After 2 hours, blood serum and liver tissues were collected and stored at −80°C for protein and RNA analysis. All experiments with mice were approved by the Animal Care Committee of the University of Ulsan, Ulsan, Korea.

### 2.4. Transfection

RAW 264.7 cells (5 × 10^5^/mL) were cultured in 6-well plates for 3 h and transfected with IL-10 siRNA (100 nM) or TN-C siRNA (100 nM) from Santa Cruz Biotechnology using Lipofectamine 2000 according to the manufacturer's instructions. After transfection, cells were incubated with or without CORM-2 (20 *μ*M) and then stimulated with or without LPS (100 ng/mL).

### 2.5. Western Blotting

Cell extracts were lysed using lysis buffer containing RIPA buffer, protease inhibitor, and phosphatase inhibitors. After lysis, protein concentration was measured by BCA assay (Pierce Biotechnology Inc., Rockford, IL, USA). Samples containing equal amounts of protein were subjected to electrophoresis and proteins were transferred to polyvinylidene difluoride (PVDF) membranes. Membranes were blocked with 5% skim milk for 20 min and then incubated at 4°C overnight with primary antibodies, followed by secondary antibodies against TN-C and *β*-actin conjugated with horseradish peroxidase. The enhanced chemiluminescence (ECL) Western Blotting Detection System (GE Healthcare Life Sciences, Buckinghamshire, UK) was used to visualize the immunoreactive bands.

### 2.6. Reverse Transcription-Polymerase Chain Reaction (RT-PCR)

Total RNA isolation was performed from RAW 264.7 macrophages using TRIzol reagent (Invitrogen) according to manufacturer's instructions. Briefly, total RNA (2 *μ*g) was used to prepare cDNA by using M-MLV reverse transcriptase (Promega Corporation, Madison, WI, USA) and oligo (dT) 15 primer (Promega). The resulted cDNA was subjected to PCR for mouse GAPDH (5′-AGGCCGGTGCTGAGTATGTC-3′, 5′-TGCCTGCTTCACCTTCT-3′, 530 bp), HO-1 (5′-TCCCAGACACCGCTCCTCCAG-3′, 5′-GGATTTGGGGCTGGTTTC-3′, 313 bp), TN-C (5′-CAGGTACTTCTTCACGGAGC-3′, 5′-GCAGTCTTCCCCAGTGAAAC-3′, 834 bp), TNF-*α* (5′-AGCCCACGTCGTAGCAAACCACCAA-3′, 5′-ACACCCATTCCCTTCACAGAGCAAT-3′, 421 bp), IL-6 (5′-GTGGAAATGAGAAAAGAGTTGT-3′, 5′-CCTCTTGGTTGAAGATATGAAT-3′, 283 bp), and IL-10 (5′-GACAATAACTGCACCCACTT-3′, 5′-TCAAATGCTCCTTGATTTCT-3′, 250 bp), and GAPDH was used as internal loading control.

### 2.7. Real Time RT-PCR

Total RNA was extracted from RAW 264.7 peritoneal macrophages/liver tissues using TRIzol reagent (Invitrogen) according to the manufacturer's instructions. In addition, cDNA was prepared by using M-MLV reverse transcriptase (Promega) and oligo (dT) 15 primer (Promega). The formulated cDNA was subjected to Real Time RT-PCR using SYBR Green qPCR Master Mix (2x) (USB products, Affymetrix) on an ABI 7500 Fast Real-Time PCR System (Applied Biosystems) for mouse GAPDH (5′-GGGAAGCCCATCACCATCT-3′, 5′-CGGCCTCACCCCATTTG-3′), TN-C (5′-ACCATGCTGAGATAGATGTTCCAAA-3′, 5′-CTTGACAGCAGAAACACCAATCC-3′), TNF-A (5′-AGACCCTCACACTCAGATCACTTTC-3′, 5′-TTGCTACGACGTGGGCTACA-3′), IL-6 (5′-CGATGATGCACTTGCAGAAA-3′, 5′-TGGAAATTGGGGTAGGAAGG-3′), IL-10 (5′-ACTGCTATGCTGCCTGCTCTTACT-3′, 5′-GAATTCAAATGCTCCTTGATTTCT-3′), and HO-1 (5′-TCAGTCCCAAACCTCGCGGT-3′, 5′-GCTGTGCAGGTGTTGAGCC-3′). GAPDH was used as internal loading control to normalize all PCR products.

### 2.8. Enzyme Linked Immunosorbent Assay (ELISA)

Macrophages on 6-well plates were incubated overnight and then pretreated with CORM-2 for 1 h followed by stimulation with LPS for 24 h. In addition, mice were administrated with CORM-2 for 2 h and then sepsis was induced by LPS injection. After 2 h, supernatants collected from various samples or blood serum collected from different mice were assayed for TNF-*α* and IL-6 by using a mouse ELISA kit (Biolegend).

### 2.9. Statistical Analysis

Statistical differences between groups were evaluated by one-way ANOVA (nonparametric) or Student's *t*-test when multiple groups were compared. Results are expressed as the means ± SEM. Differences were considered to be significant when ^*∗*^ *P* < 0.05, ^*∗∗*^ *P* < 0.01, and ^*∗∗∗*^ *P* < 0.001.

## 3. Results

### 3.1. LPS Increases TN-C Expression in a Time- and Dose-Dependent Manner

**Figure 1:**
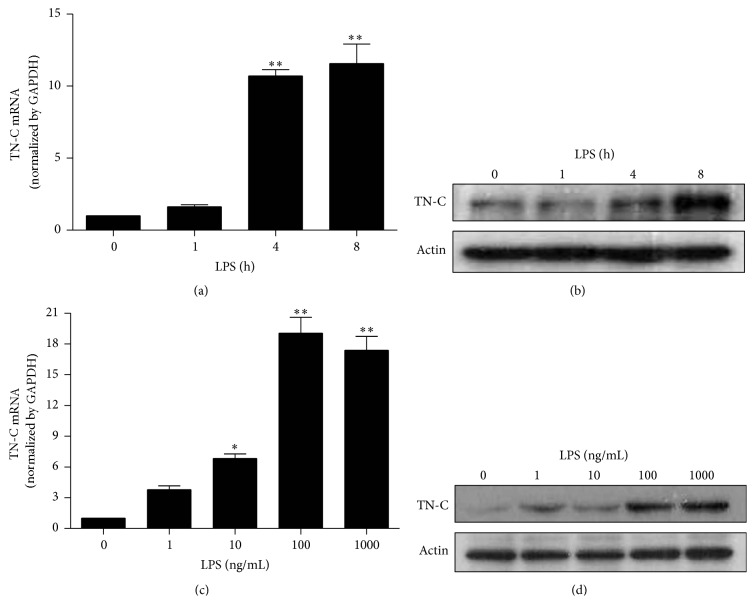
LPS increases TN-C expression in a time- and dose-dependent manner in RAW 264.7 macrophages. Cells were treated with 100 ng/mL LPS for 0, 1, 4, and 8 h and (a) mRNA expression of TN-C was detected by Real Time RT-PCR and (b) TN-C protein expression was measured by Western blotting. Cells were treated with LPS for 8 h (0, 1, 10, 100, and 1000 ng/mL). Harvested cells were subjected to (c) mRNA and (d) protein analysis for TN-C. Representative bands are shown. Data represents mean ± SEM, ^*∗*^ *P* < 0.05, and ^*∗∗*^ *P* < 0.001 as compared with control.

Macrophages have pro- or anti-inflammatory functions depending on the type of stimuli [22]. Stimulation of macrophages with Gram-negative bacterial LPS can enhance the expression of TN-C [7]. TLR4 was involved in the induction of TN-C and subsequent cytokine synthesis in both human and murine macrophages [23] and human chondrocytes [24]. In the present study, we examined inflammatory responses in murine RAW 264.7 macrophages treated with LPS (100 ng/mL). TN-C mRNA and protein expression increased at 4 and 8 h after LPS treatment (Figures 1(a) and 1(b)), respectively. Therefore, in subsequent experiments, we measured TN-C mRNA and protein expression at 8 h. Furthermore, LPS dose-dependently increased TN-C mRNA and protein expression at 8 h (Figures 1(c) and 1(d)). These results suggest that LPS induces TN-C expression in a time- and dose-dependent manner in RAW 264.7 macrophages.

### 3.2. CO Inhibits LPS-Induced TN-C Expression and Proinflammatory Cytokines Production

**Figure 2:**
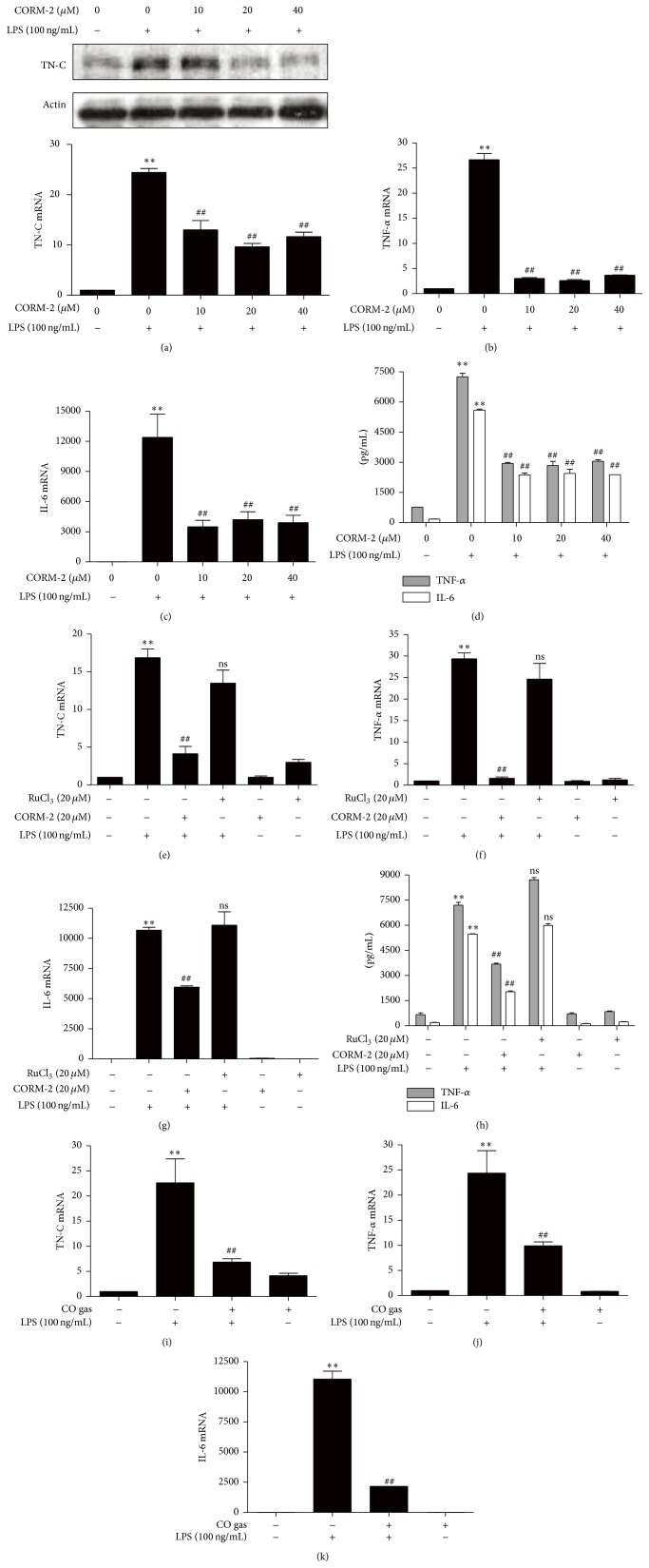
CO inhibits LPS-induced TN-C expression and proinflammatory cytokines in RAW 264.7 macrophages. (a–d) To detect the TN-C expression and proinflammatory cytokine production, macrophages were pretreated with CORM-2 (0, 10, 20, and 40 *μ*M) for 1 h and then stimulated with LPS (100 ng/mL) for 8 h. The protein levels (upper) and mRNA levels (lower) of TN-C were measured by Western blotting and Real Time RT-PCR. The mRNA levels (b, c) and protein levels (d) of TNF-*α* and IL-6 were reduced by CORM-2. The amount of mRNA with Real Time RT-PCR was detected and protein expression was measured with ELISA after 24 h of LPS treatment. (e–h) Cells were pretreated with CORM-2 (20 *μ*M) or RuCl_3_ (20 *μ*M) for 1 h followed by stimulation in the presence or absence of LPS (100 ng/mL) for 8 h. RuCl_3_ was used as a negative control for CORM-2. The levels of mRNA in TN-C (e), TNF-*α* (f), and IL-6 (g) after treatment of CORM-2 were measured by Real Time RT-PCR and the protein levels of TNF-*α* and IL-6 were detected by ELISA after 24 h of LPS treatment (h). (i–k) Cells were pretreated with CO gas (250 ppm) for 2 h and incubated with LPS (100 ng/mL) stimulated for 8 h. TN-C (i), TNF-*α* (j), and IL-6 (k) mRNA levels were detected by Real Time RT-PCR. Data represents mean ± SEM, ^*∗∗*^ *P* < 0.001 as compared with control; ^##^ *P* < 0.001 and ns, nonsignificant, as compared with the cells exposed to LPS alone.

The anti-inflammatory, antiapoptotic, and cytoprotective properties of CO are well known [25]. Furthermore, it has been reported that the generation of endogenous CO was necessary for IL-10-dependent inhibition of TNF-*α* expression [20]. CO can be generated pharmacologically from CO-releasing molecules (CORMs), which consist of a heavy metal such as ruthenium surrounded by carbonyl groups [26, 27]. In brain endothelial cells, the LPS-induced activation of inflammatory signals such as NF-*κ*B (p65), COX-2 expression, and PGE2 production was inhibited by CORM-2 pretreatment [28]. In the present study, we investigated the effects of CO on TN-C-mediated inflammation. RAW 264.7 cells were pretreated with CORM-2 and then stimulated with LPS. We found that CORM-2 significantly and dose-dependently suppressed the expression of LPS-stimulated TN-C expression (Figure 2(a)). In addition, pretreatment with CORM-2 significantly reduced the mRNA and protein levels of proinflammatory cytokines such as TNF-*α* and IL-6 (Figures 2(b), 2(c), and 2(d)), respectively. To confirm the effects of CO on TN-C-meditated inflammation, cells were pretreated with or without CORM-2 or RuCl_3_ (negative control for CORM-2) and then stimulated with or without LPS. Interestingly, CORM-2 significantly downregulated LPS-induced TN-C as well as proinflammatory cytokines expression whereas RuCl_3_ did not have any effect (Figures 2(e), 2(f), 2(g), and 2(h)). To confirm the effects of CO, we pretreated the cells with CO gas and then incubated them with LPS. Consistently, CO gas dramatically reduced LPS-stimulated expression of TN-C (Figure 2(i)), as well as TNF-*α* (Figure 2(j)) and IL-6 (Figure 2(k)).

**Figure 3:**
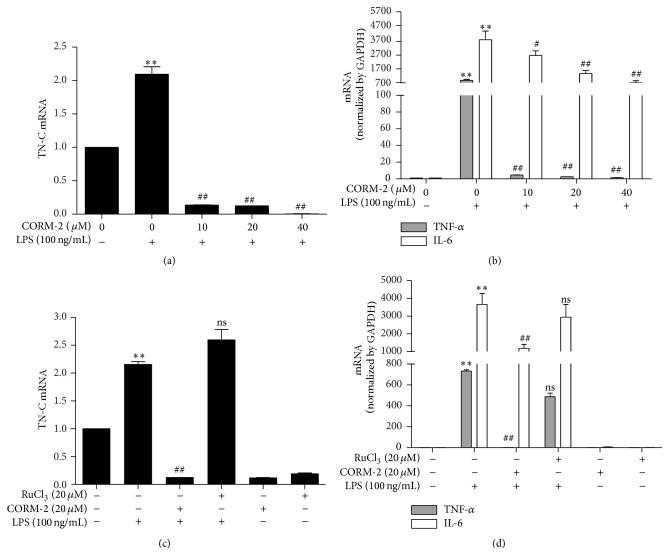
CO inhibits LPS-induced TN-C expression and proinflammatory cytokines in peritoneal macrophages. (a-b) Macrophages were pretreated with CORM-2 (0, 10, 20, and 40 *μ*M) for 1 h and then stimulated with LPS (100 ng/mL) for 8 h. TN-C, TNF-*α*, and IL-6 mRNA levels were detected by Real Time RT-PCR, respectively. (c-d) Cells were pretreated with CORM-2 (20 *μ*M) or RuCl_3_ (20 *μ*M) for 1 h and stimulated with or without LPS (100 ng/mL) for 8 h. RuCl_3_ was used as a negative control for CORM-2. TN-C, TNF-*α*, and IL-6 mRNA levels were detected by Real Time RT-PCR. Data represents mean ± SEM, ^*∗∗*^ *P* < 0.001 as compared with control; ^#^ *P* < 0.05, ^##^ *P* < 0.001, and ns, nonsignificant, as compared with the cells exposed to LPS alone.

To further confirm the effects of CO on LPS-induced TN-C expression and proinflammatory cytokines, mouse peritoneal macrophages were pretreated with CORM-2 at various concentrations and incubated with LPS. We found that CORM-2 dramatically decreased LPS-induced TN-C (Figure 3(a)) and its downstream cytokines (Figure 3(b)). In contrast, RuCl_3_ did not reduce the expression of TN-C and proinflammatory cytokines (Figures 3(c) and 3(d)). These results suggest that CO can suppress LPS-mediated inflammatory signals* in vitro*.

### 3.3. CO/HO-1 Inhibits TN-C-Mediated Inflammation via IL-10 Induction

**Figure 4:**
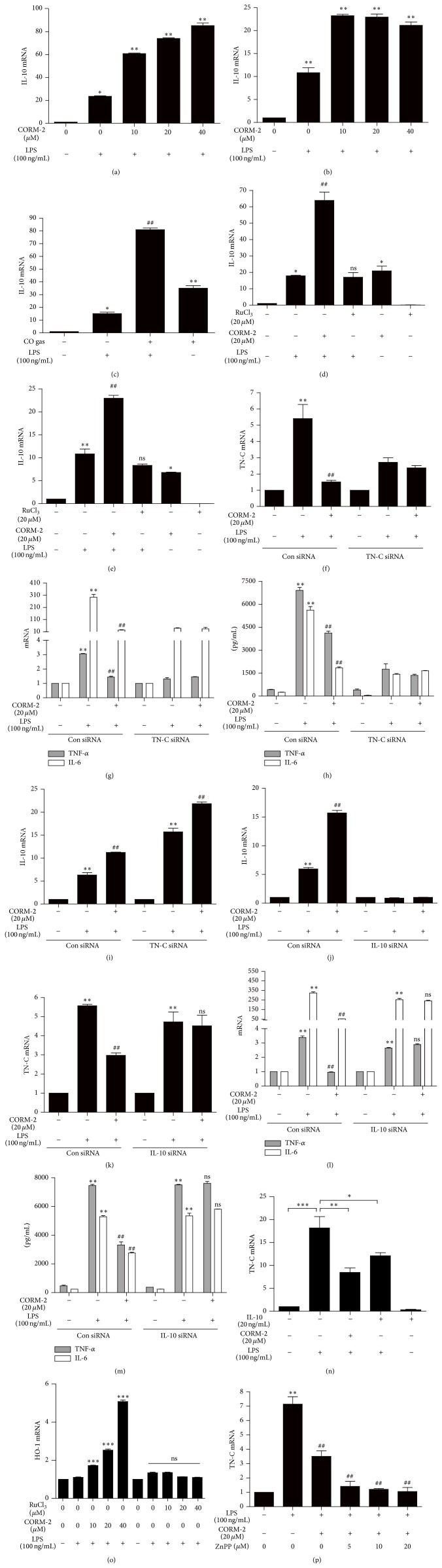
CO inhibits TN-C-mediated inflammation* via* IL-10 induction in RAW 264.7 macrophages and peritoneal macrophages. (a-b) Cells were pretreated with CORM-2 (0, 10, 20, and 40 *μ*M) for 1 h and then stimulated with LPS (100 ng/mL) for 8 h. IL-10 mRNA level was detected by Real Time RT-PCR in RAW 264.7 (a) and peritoneal macrophages (b). After treatment of CO gas (250 ppm) for 2 h and being incubated with LPS (100 ng/mL) stimulated for 8 h, IL-10 mRNA level was detected by Real Time RT-PCR (c). (d-e) Cells with CORM-2 (20 *μ*M) or RuCl_3_ (20 *μ*M) for 1 h were stimulated with LPS (100 ng/mL) for 8 h. IL-10 mRNA levels increased with CORM-2 which was detected by Real Time RT-PCR in RAW 264.7 (d) and peritoneal macrophages (e). RuCl_3_ was used as a negative control for CORM-2. (f–i) RAW 264.7 cells were transfected with TN-C siRNA or control siRNA (scRNA). After treatment with CORM-2 (20 *μ*M) for 1 h, cells were stimulated with 100 ng/mL LPS for 8 h to evaluate mRNA levels of TN-C (f) and TNF-*α* and IL-6 (g) by Real Time RT-PCR. The protein levels of TNF-*α* and IL-6 were measured by ELISA (h) after 24 h LPS treatment. The mRNA levels of IL-10 were detected by Real Time RT-PCR (i). (j–m) RAW 264.7 macrophages were transfected with IL-10 siRNA or control siRNA (scRNA). After treatment with CORM-2 (20 *μ*M) for 1 h, cells were stimulated with 100 ng/mL LPS for 8 h. The mRNA levels of IL-10 (j), TN-C (k), and TNF-*α* and IL-6 (l) were detected by Real Time RT-PCR, and protein levels of TNF-*α* and IL-6 were measured by ELISA (m) after 24 h LPS treatment. RAW 264.7 cells were pretreated with CORM2 (20 *μ*M) or mouse recombinant IL-10 (20 ng/mL) for 1 h followed by the stimulation of LPS (100 ng/mL) for another 8 h. TN-C mRNA levels were determined by Real Time PCR (n). (o-p) RAW 264.7 cells were pretreated with CORM2 or RuCl_3_ in various concentrations (0, 10, 20, and 40 *μ*M) with or without ZnPP (0, 5, 10, and 20 *μ*M) followed by 8 h LPS treatment. The mRNA levels of HO-1 (o) and TN-C (p) were detected by Real Time PCR. Data represents mean ± SEM, ^*∗*^ *P* < 0.05, and ^*∗∗*^ *P* < 0.001 as compared with control; ^#^ *P* < 0.05, ^##^ *P* < 0.001, and ns, nonsignificant, as compared with the cells exposed to LPS alone (normal condition or TN-C/IL-10 siRNA, compared separately).

Low doses of CO suppressed inflammatory responses in a murine model of sepsis through inhibition of inflammatory cytokines production [18, 29] as well as increased LPS-induced expression of the anti-inflammatory cytokine IL-10 in various cell types [19, 29]. In addition, mice deficient with TN-C displayed lower levels of TNF-*α* and downstream cytokine production in LPS-treated septic mice and bone marrow-derived macrophages (BMDMs) [8]. In our study, we investigated the effects of CO on TN-C-induced proinflammatory cytokines expression and the expression of anti-inflammatory IL-10 in RAW 264.7 and peritoneal macrophages. CORM-2 significantly and dose-dependently induced the expression of IL-10 in LPS-stimulated macrophages (Figures 4(a) and 4(b)). Furthermore, treatment with CO gas significantly increased levels of IL-10 (Figure 4(c)) in LPS-stimulated RAW 264.7 macrophages. However, treatment with RuCl_3_ did not have any effect on IL-10 expression in these cells (Figures 4(d) and 4(e)). These observations indicate that the anti-inflammatory effects of CO are mediated by IL-10 in LPS-stimulated macrophages. The incubation of RAW 264.7 cells with TN-C siRNA significantly suppressed the effects of LPS on TN-C and proinflammatory cytokines production (Figures 4(f), 4(g), and 4(h)), whereas it had no effect on IL-10 expression (Figure 4(i)), suggesting that IL-10 is regulated independently of TN-C and its downstream cytokines. To confirm the function of CO-induced IL-10 on TN-C-mediated inflammation, macrophages were transfected with IL-10 siRNA and treated with CORM-2 prior to LPS-stimulation. The efficiency of IL-10 siRNA transfection was shown in Figure 4(j). We found that IL-10 siRNA reversed the inhibitory effect of CORM-2 on TN-C expression and inflammatory cytokines production in LPS-stimulated macrophages relative to control siRNA (Figures 4(k), 4(l), and 4(m)). Pretreatment of recombinant IL-10 with or without LPS stimulation showed the same efficiency of CORM-2 to significantly decrease TN-C expression (Figure 4(n)). Also, HO-1 increases IL-10 production [30]. According to Inoue and colleagues [30], overexpressions of HO-1 provide the cytoprotection via the mediation of IL-10 production. Thus, we examined the expression of anti-inflammatory gene HO-1 under these conditions. Interestingly, we found that CORM-2 significantly increased the level of HO-1 expression (Figure 4(o)), whereas RuCl_3_ did not have any effect on HO-1 expression (Figure 4(o)) and conversely decreased the expression levels of TN-C. The inhibition of HO activity using ZnPPIX, however, did not reverse the effects of CORM-2 on TN-C expression (Figure 4(p)) indicating that CORM-2 mediated suppression of TN-C is independent of HO activity in LPS-stimulated RAW 264.7 macrophages. Based on these results, we conclude that CO-induced IL-10 inhibits TN-C-mediated inflammation.

### 3.4. CO Inhibits TN-C-Mediated Inflammation* In Vivo* in a Septic Mice Model

**Figure 5:**
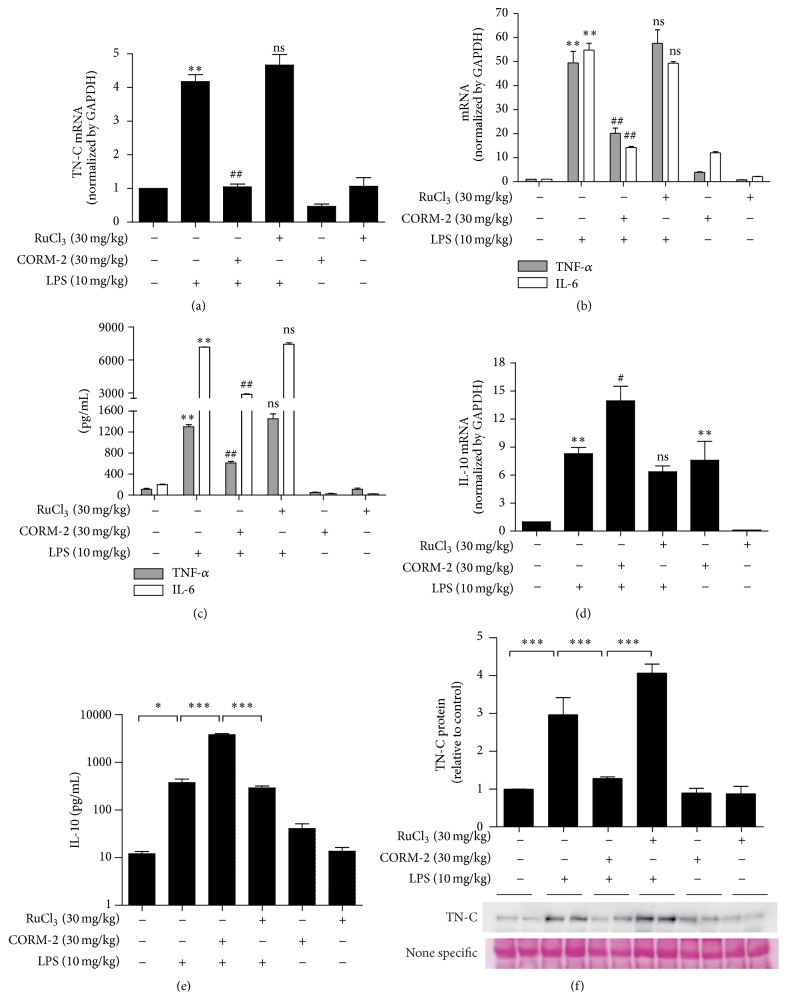
CO inhibits TN-C-mediated inflammation a septic mouse model. Wild type seven-week-old male C57BL/6 mice were pretreated with CORM-2 (30 mg/kg, i.p.) or RuCl_3_ (30 mg/kg, i.p.) for 2 h and then mice were injected with LPS (10 mg/kg, i.p.) for 2 h. Liver tissues were analyzed for mRNA levels of TN-C (a) and TNF-*α* and IL-6 (b) by Real Time RT-PCR. Blood serum was analyzed for protein expression of TNF-*α*, IL-6 (c). IL-10 mRNA (d) by Real Time RT-PCR and IL-10 protein (e) by ELISA were measured. The protein levels of TN-C from serum were measured by Western blotting (f). Loading control was detected by ponceau staining. The relative signal intensity of bands was determined and standardized using ImageJ software. Data represents mean ± SEM, ^*∗*^ *P* < 0.05, and ^*∗∗*^ *P* < 0.001 when compared with control; ^#^ *P* < 0.05, ^##^ *P* < 0.001, and ns, nonsignificant, as compared with the cells exposed to LPS alone.

**Figure 6:**
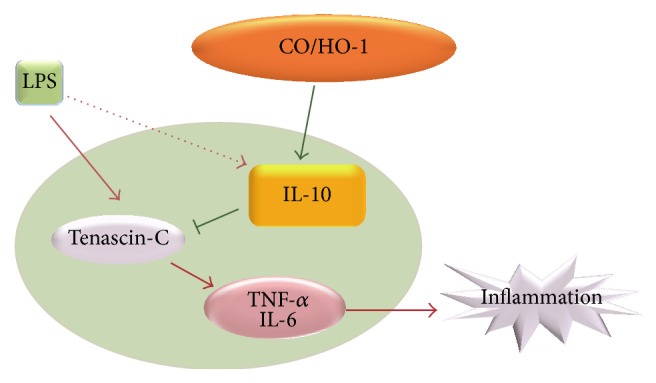
Schemata of the proposed CO/IL-10-dependent anti-inflammatory signaling pathway. Carbon monoxide increased IL-10 expression and inhibited TN-C-induced inflammation in a septic mouse model* in vivo* and* in vitro*.

Sepsis, a systemic inflammatory response, results from excessive production of proinflammatory cytokines by LPS stimulation [31]. In addition, proinflammatory cytokines such as TNF-*α*, IL-1*β*, and IL-6 have been found at higher levels in septic patients [32, 33]. Administration of LPS in mice revealed that TN-C expression is necessary for proinflammatory signaling [8]. Furthermore, application of exogenous CO inhibits LPS-induced production of TNF-*α* while it increases IL-10 production* in vitro* and* in vivo* [29]. However, there are no reports regarding the effects of CO-mediated IL-10 production in relation to the regulation of TN-C and inflammation in a septic mouse model. In our study, to examine the* in vivo* effects of CO using CORM-2 on LPS-induced endotoxemia and TN-C-mediated inflammatory cytokines expression, we pretreated mice with CORM-2 (30 mg/kg, i.p.) or RuCl_3_ (30 mg/kg, i.p.) for 2 h and LPS (10 mg/kg, i.p.) for 2 h. Interestingly, CORM-2 significantly decreased TN-C (Figure 5(a)), TNF-*α* and IL-6 mRNA expression (Figure 5(b)), and protein secretion (Figure 5(c)) and simultaneously increased IL-10 expression (Figure 5(d)) in liver tissue from LPS-induced endotoxemic mice. Also, the levels of IL-10 (Figure 5(e)) were increased and reversely TN-C levels (Figure 5(f)) were decreased in the serum of mice treated with CORM-2. Therefore, the results from* in vivo* experiments suggest that CO inhibited TN-C and its downstream inflammatory cytokines whereby IL-10 expression was upregulated in a septic mice model. A scheme is provided to illustrate each of these results (Figure 6).

## 4. Discussion

TN-C is unique in its distinct pattern of expression. Upon tissue injury TN-C is transiently expressed, whereas its expression is reduced after the tissue is repaired [1]. Moreover, persistent TN-C expression occurs during chronic inflammation [34]. In addition, TN-C is absent in most healthy adult tissues [2] whereas high levels are found during infection and in patients with sepsis. However, TN-C is expressed at sites of inflammation regardless of the location or type of causative insult, indicating its capability to participate in the global inflammatory response.

TN-C can increase the synthesis of cytokines in human chondrocytes [24] and myeloid cells [7] in a TLR4-dependent manner and also activate murine myeloid cells [35, 36]. Additionally, TN-C expression is transiently induced by LPS in innate immune cells in a NF-*κ*B-dependent manner [7, 37] and its dysregulation is observed in both autoimmune and inflammatory diseases, such as sepsis [38]. In the present study, we found that LPS significantly increased TN-C and proinflammatory cytokines production in macrophages as well as in a mouse model. Therefore, understanding which compounds can inhibit TLR-mediated TN-C expression and cytokines production may refine strategies to manipulate excessive inflammation.

Recently, researchers reported that CO gas can exert beneficial effects in various cell and animal models. CO plays an important role in preventing apoptosis in several cell types such as endothelial cells [39], fibroblasts [40], and pancreatic *β*-cells [41] and inhibits the proliferation of smooth muscle cells [42], thus preventing atherosclerotic lesions. In animal models, CO reduced graft rejection [43] and lung inflammation [44].

CORM compounds provide a reliable source of CO that can mimic CO gas in many biological functions [26, 45]. Therefore, CORMs represent important tools to understand the biological significance of CO in physiology and disease. CORM-2 was the first compound used to deliver CO in biological systems in a controlled manner [27]. In the current study, to examine the effects of CO, we pretreated macrophages and mice with CORM-2 in an LPS-stimulated inflammation model. Interestingly, CORM-2 significantly decreased LPS-induced TN-C and proinflammatory cytokines production* in vitro* and* in vivo*. Similarly, pretreatment with CO gas significantly decreased LPS-stimulated TN-C and cytokines production in macrophages, supporting the direct effects of CO on TN-C-mediated inflammation. In addition, RuCl_3,_ a negative control for CORM-2, did not affect LPS-mediated TN-C, TNF-*α*, and IL-6 expression in macrophages or in septic mice. These results confirm that CO inhibits LPS-mediated TN-C and proinflammatory cytokines production.

The anti-inflammatory cytokine IL-10 plays a crucial role in dampening Toll-like receptor (TLR) signaling-induced proinflammatory genes. Interestingly, CO was found to increase the levels of anti-inflammatory, IL-10 [19], while the levels of proinflammatory cytokines were decreased in several* in vitro* systems [18, 29]. Additionally, CORM-2 was also found to regulate inflammatory responses through decreasing IL-1*β* expression and increasing IL-10 expression [46]. In a sepsis model, CO-mediated activation of the MKK3/p38 MAPK signaling pathway was involved in the induction of IL-10 [29]. In our investigation, we determined that the effects of CO significantly increased IL-10 expression under LPS-stimulated conditions while RuCl_3_ had no effect* in vitro* or* in vivo*. Furthermore, IL-10 siRNA significantly reversed the effects of CORM-2 on TN-C and proinflammatory cytokines production whereas TN-C siRNA significantly decreased proinflammatory gene expression levels without having an effect on CO-mediated IL-10 expression. This evidence suggests that CO-mediated IL-10 expression was involved in inhibition of TN-C-mediated inflammation.

In summary, we identified that CO-induced IL-10 was involved in the inhibition of TLR4 signaling-dependent TN-C expression and thus inhibited the inflammatory response* in vitro* and* in vivo*. This study describes a novel CO-dependent IL-10 signaling pathway responsible for the inhibition of TN-C-driven inflammation and potentially provides the rationale for novel therapeutic strategies for the treatment of inflammatory diseases.

## Abbreviations

CO: Carbon monoxide
CORM-2: CO-releasing molecule-2
TNF: Tumor necrosis factor
ECL: Enhanced chemiluminescence
IL: Interleukin
TN-C: Tenascin-C
DAMPs: Damage-associated molecular patterns
ECM: Extracellular matrix
TLR: Toll-like receptor.

## References

[1] Midwood K. S., Hussenet T., Langlois B., Orend G. (2011). Advances in tenascin-C biology. *Cellular and Molecular Life Sciences*.

[2] Chiquet-Ehrismann R., Chiquet M. (2003). Tenascins: regulation and putative functions during pathological stress. *Journal of Pathology*.

[3] Kaarteenaho-Wiik R., Lakari E., Soini Y., Pollanen R., Kinnula V. L., Paakko P. (2000). Tenascin expression and distribution in pleural inflammatory and fibrotic diseases. *Journal of Histochemistry and Cytochemistry*.

[4] Päällysaho T., Tervo K., Kivelä T., Virtanen I., Tarkkanen A., Tervo T. (1993). Cellular fibronectin and tenascin in an orbital nylon prosthesis removed because of infection caused by *Staphylococcus aureus*. *Graefe's Archive for Clinical and Experimental Ophthalmology*.

[5] Schenk S., Muser J., Vollmer G., Chiquet-Ehrismann R. (1995). Tenascin-C in serum: a questionable tumor marker. *International Journal of Cancer*.

[6] Midwood K., Sacre S., Piccinini A. M. (2009). Tenascin-C is an endogenous activator of Toll-like receptor 4 that is essential for maintaining inflammation in arthritic joint disease. *Nature Medicine*.

[7] Goh F. G., Piccinini A. M., Krausgruber T., Udalova I. A., Midwood K. S. (2010). Transcriptional regulation of the endogenous danger signal tenascin-C: a novel autocrine loop in inflammation. *Journal of Immunology*.

[8] Piccinini A. M., Midwood K. S. (2012). Endogenous control of immunity against infection: tenascin-C regulates TLR4-mediated inflammation via microRNA-155. *Cell Reports*.

[9] Ekblom M., Fassler R., Tomasini-Johansson B., Nilsson K., Ekblom P. (1993). Downregulation of tenascin expression by glucocorticoids in bone marrow stromal cells and in fibroblasts. *Journal of Cell Biology*.

[10] Murray P. J. (2006). Understanding and exploiting the endogenous interleukin-10/STAT3-mediated anti-inflammatory response. *Current Opinion in Pharmacology*.

[11] O'Garra A., Barrat F. J., Castro A. G., Vicari A., Hawrylowicz C. (2008). Strategies for use of IL-10 or its antagonists in human disease. *Immunological Reviews*.

[12] Asadullah K., Sterry W., Volk H. D. (2003). Interleukin-10 therapy—review of a new approach. *Pharmacological Reviews*.

[13] Murray P. J. (2006). STAT3-mediated anti-inflammatory signalling. *Biochemical Society Transactions*.

[14] Ryter S. W., Otterbein L. E. (2004). Carbon monoxide in biology and medicine. *BioEssays*.

[15] Otterbein L. E., Kolls J. K., Mantell L. L., Cook J. L., Alam J., Choi A. M. K. (1999). Exogenous administration of heme oxygenase-1 by gene transfer provides protection against hyperoxia-induced lung injury. *Journal of Clinical Investigation*.

[16] Soares M. P., Lin Y., Anrather J. (1998). Expression of heme oxygenase-1 can determine cardiac xenograft survival. *Nature Medicine*.

[17] Sawle P., Foresti R., Mann B. E., Johnson T. R., Green C. J., Motterlini R. (2005). Carbon monoxide-releasing molecules (CO-RMs) attenuate the inflammatory response elicited by lipopolysaccharide in RAW264.7 murine macrophages. *British Journal of Pharmacology*.

[18] Morse D., Pischke S. E., Zhou Z. (2003). Suppression of inflammatory cytokine production by carbon monoxide involves the JNK pathway and AP-1. *The Journal of Biological Chemistry*.

[19] Lee T.-S., Chau L.-Y. (2002). Heme oxygenase-1 mediates the anti-inflammatory effect of interleukin-10 in mice. *Nature Medicine*.

[20] Yachie A., Niida Y., Wada T. (1999). Oxidative stress causes enhanced endothelial cell injury in human heme oxygenase-1 deficiency. *Journal of Clinical Investigation*.

[21] Tsoyi K., Jang H. J., Kim J. W. (2011). Stimulation of Alpha7 nicotinic acetylcholine receptor by nicotine attenuates inflammatory response in macrophages and improves survival in experimental model of sepsis through heme oxygenase-1 induction. *Antioxidants and Redox Signaling*.

[22] Martinez F. O., Sica A., Mantovani A., Locati M. (2008). Macrophage activation and polarization. *Frontiers in Bioscience*.

[23] Midwood K. S., Orend G. (2009). The role of tenascin-C in tissue injury and tumorigenesis. *Journal of Cell Communication and Signaling*.

[24] Patel L., Sun W., Glasson S. S., Morris E. A., Flannery C. R., Chockalingam P. S. (2011). Tenascin-C induces inflammatory mediators and matrix degradation in osteoarthritic cartilage. *BMC Musculoskeletal Disorders*.

[25] Ryter S. W., Choi A. M. (2006). Therapeutic applications of carbon monoxide in lung disease. *Current Opinion in Pharmacology*.

[26] Motterlini R. (2007). Carbon monoxide-releasing molecules (CO-RMs): vasodilatory, anti-ischaemic and anti-inflammatory activities. *Biochemical Society Transactions*.

[27] Motterlini R., Clark J. E., Foresti R., Sarathchandra P., Mann B. E., Green C. J. (2002). Carbon monoxide-releasing molecules: characterization of biochemical and vascular activities. *Circulation research*.

[28] Shih R.-H., Yang C.-M. (2010). Induction of heme oxygenase-1 attenuates lipopolysaccharide-induced cyclooxygenase-2 expression in mouse brain endothelial cells. *Journal of Neuroinflammation*.

[29] Otterbein L. E., Bach F. H., Alam J. (2000). Carbon monoxide has anti-inflammatory effects involving the mitogen- activated protein kinase pathway. *Nature Medicine*.

[30] Inoue S., Suzuki M., Nagashima Y. (2001). Transfer of heme oxygenase 1 cDNA by a replication-deficient adenovirus enhances interleukin 10 production from alveolar macrophages that attenuates lipopolysaccharide-induced acute lung injury in mice. *Human Gene Therapy*.

[31] Oberholzer A., Oberholzer C., Moldawer L. L. (2001). Sepsis syndromes: understanding the role of innate and acquired immunity. *Shock*.

[32] Arnalich F., Garcia-Palomero E., López J. (2000). Predictive value of nuclear factor kappaB activity and plasma cytokine levels in patients with sepsis. *Infection and Immunity*.

[33] van der Poll T., van Deventer S. J. H. (1999). Cytokines and anticytokines in the pathogenesis of sepsis. *Infectious Disease Clinics of North America*.

[34] Udalova I. A., Ruhmann M., Thomson S. J. P., Midwood K. S. (2011). Expression and immune function of tenascin-C. *Critical Reviews in Immunology*.

[35] Kanayama M., Kurotaki D., Morimoto J. (2009). *α*9 integrin and its ligands constitute critical joint microenvironments for development of autoimmune arthritis. *Journal of Immunology*.

[36] Kanayama M., Morimoto J., Matsui Y. (2011). *α*9*β*1 integrin-mediated signaling serves as an intrinsic regulator of pathogenic Th17 cell generation. *Journal of Immunology*.

[37] Bala S., Marcos M., Kodys K. (2011). Up-regulation of microRNA-155 in macrophages contributes to increased tumor necrosis factor *α* (TNF*α*) production via increased mRNA half-life in alcoholic liver disease. *The Journal of Biological Chemistry*.

[38] Tili E., Michaille J.-J., Cimino A. (2007). Modulation of miR-155 and miR-125b levels following lipopolysaccharide/TNF-alpha stimulation and their possible roles in regulating the response to endotoxin shock. *Journal of Immunology*.

[39] Wang X., Wang Y., Kim H. P., Nakahira K., Ryter S. W., Choi A. M. K. (2007). Carbon monoxide protects against hyperoxia-induced endothelial cell apoptosis by inhibiting reactive oxygen species formation. *Journal of Biological Chemistry*.

[40] Petrache I., Otterbein L. E., Alam J., Wiegand G. W., Choi A. M. K. (2000). Heme oxygenase-1 inhibits TNF-alpha-induced apoptosis in cultured fibroblasts. *The American Journal of Physiology—Lung Cellular and Molecular Physiology*.

[41] Günther L., Berberat P. O., Haga M. (2002). Carbon monoxide protects pancreatic *β*-cells from apoptosis and improves islet function/survival after transplantation. *Diabetes*.

[42] Togane Y., Morita T., Suematsu M., Ishimura Y., Yamazaki J.-I., Katayama S. (2000). Protective roles of endogenous carbon monoxide in neointimal development elicited by arterial injury. *American Journal of Physiology: Heart and Circulatory Physiology*.

[43] Nakao A., Choi A. M. K., Murase N. (2006). Protective effect of carbon monoxide in transplantation. *Journal of Cellular and Molecular Medicine*.

[44] Ryter S. W., Alam J., Choi A. M. K. (2006). Heme oxygenase-1/carbon monoxide: from basic science to therapeutic applications. *Physiological Reviews*.

[45] Motterlini R., Mann B. E., Johnson T. R., Clark J. E., Foresti R., Green C. J. (2003). Bioactivity and pharmacological actions of carbon monoxide-releasing molecules. *Current Pharmaceutical Design*.

[46] Lin L.-C., Ho F.-M., Yen S.-J. (2010). Carbon monoxide induces cyclooxygenase-2 expression through MAPKs and PKG in phagocytes. *International Immunopharmacology*.

